# The Relationships among Proactive Personality, Work Engagement, and Perceived Work Competence in Sports Coaches: The Moderating Role of Perceived Supervisor Support

**DOI:** 10.3390/ijerph191912707

**Published:** 2022-10-04

**Authors:** Shin-Huei Lin, Wan-Chen Lu, Yi-Chieh Chen, Ming-Han Wu

**Affiliations:** 1 Department of Leisure Management, National Pingtung University, Pingtung City 900392, Taiwan; 2 Department of Athletics, National Taiwan University, Taipei 10617, Taiwan

**Keywords:** proactive personality, work engagement, supervisor support, perceived work competence, job demands-resources model

## Abstract

Grounded in the job demands-resources model, this study examines the moderating role of supervisor support and the mediating role of sports coaches’ work engagement in the relationship between proactive personality and perceived work competence. A total of 261 school sports coaches in Taiwan participated in the study. The results indicated that work engagement positively mediates the relationship between sports coaches’ proactive personality and perceived work competence. Separately, supervisor support weakens the link between proactive personality and work engagement but strengthens the relationship between work engagement and perceived work competence; however, taken together, supervisor support weakens the indirect effects of proactive personality on perceived work competence through job engagement. Under the boundary condition of perceived supervisor support, the sports coaches’ proactive personality is a critical antecedent of perceived work competence through work engagement. We suggest that proactive sports coaches are assets for schools because they possess the drive and energy for self-improvement, promoting organizational progress automatically.

## 1. Introduction

School coaching plays a vital role in the development of student-athletes [1]. They not only formulate training sessions and strategies to improve athletes’ performance [2] but also influence athletes’ self-perceptions toward sports [3]. As sports competitions have become increasingly complex and demanding over the past decades, coaches now need more competencies to respond to the changing environment [4]. However, in the field of sports psychology, only a few studies have been conducted on enhancing coaches’ work competencies [3]. Therefore, in the current study, we assess proactive personality as well as work engagement to predict coaches’ relevant work competencies from the perspective of the job demands-resources (JD-R) model. 

Perceived work competence, i.e., individuals’ evaluations of their capabilities to execute particular tasks or perform in specific domains [5], has been demonstrated to lead to better job performance [6,7] and well-being [8]. To address the concern of enhancing employees’ perception of work competence, a number of studies have indicated that personality traits are positively correlated with perceived work competence, e.g., optimism [9] and proactive personality [10,11]. For employees with proactive personalities, previous studies have indicated that their efforts aimed at achieving work goals improve their perceived work competence [11], which promotes mastery and confidence and allows employees to grow their abilities [10].

A proactive personality is a “stable disposition to take personal initiative in a broad range of activities and situations” [12]. Researchers have suggested that psychological conditions are a strong motivator of employees’ engagement at work [13]. Abundant research on proactive personality [14] stems from the substantial impact these personality inclinations have on work attitudes, which can be explained by job engagement. In the organizational context, the widely recognized JD-R model has underlined the motivation process of job-related resources, which posits that job resources (e.g., supervisor support) are especially salient for employees’ state of mind, i.e., work engagement [15]. In addition, researchers speculate that employees with more engagement at work are more likely to better harness their capabilities, knowledge, and experiences, which may lead them to have a higher perception of work competence [16]. However, it is uncertain whether the JD-R model remains robust when perceived work competence is contained as a consequence, given that perceived work competence is dissimilar to the typical outcomes of the JD-R model.

Concerning job resources, supervisor support provides a risk-free climate in which employees feel that they are unconstrained, trusted, and valued [17], and they can acquire instrumental resources, which are likely to improve their work engagement by increasing their motivation [18]. It has been demonstrated that work outcomes and employee well-being are fueled by the perception of work competence through work motivation [19]. In this vein, researchers have also suggested that individuals feel more competent when they have an opportunity to utilize their abilities and skills [20]. Hence, for employees in the motivation process, a supportive environment tends to offer opportunities to grow and develop work competencies [21], which empowers them to raise the degree of control and impact the tasks at hand; therefore, in turn, advancing their perceived work competence [22] and activating the proactive trait to take the initiative at work. Put together, through the lens of the JD-R model, we aimed to examine whether employees with a proactive personality exhibit higher work engagement and higher perceptions of work competence contingent on the level of their supervisor support.

**Figure 1:**
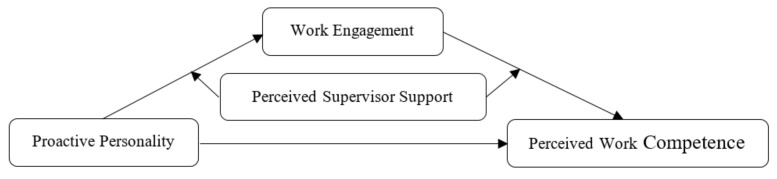
Hypothesized research model in this study.

Different from past research [23], we further adopt contextual moderators such as perceived supervisor support to examine the interaction effect on the above relationships. With this conceptual framework, our findings may contribute to the further understanding and promotion of sports coaches’ work competencies, and provide boundary conditions when interpreting the JD-R model. By adopting the well-established JD-R model, the aim of this study is to shed light on the motivation mechanism of the antecedent and non-traditional consequences of work engagement among school sports coaches. More specifically, we examined the mediating role of school sports coaches’ job engagement in the relationship between their proactive personality, perceived work competence, and the boundary condition of supervisor support on the indirect paths of the mediation model, as shown in Figure 1.

### 1.1. Job Demands-Resources Model

Schaufeli et al. [24] elaborated on the term work engagement by providing a definition to highlight vigor, dedication, and absorption as characteristics of people’s psychological states of engagement at work. Then, Bakker and Demerouti [25] framed the dual process of the JD-R model by proposing the antecedents of work engagement, i.e., job demands with motivation processes and job resources with energetic processes. Job demands comprise substantial physical and/or psychological effort and the cost of achieving job requirements; job resources mitigate job demands and promote individual fulfillment of job requirements [15]. This study focused on the motivational process of job resources within the JD-R model. Specifically, with personal resources as the antecedent (i.e., proactive personality) and the boundary condition (i.e., supervisor support), we elaborated on the motivation process of job resources to better understand whether the psychological mechanism underlying the relationship between proactive personality and perceived work competence through work engagement is influenced by supervisor support among school sports coaches.

### 1.2. Proactive Personality and Perceived Work Competence

The proactive personality has been defined as being “relatively unconstrained by situational forces, and who effects environmental change” [26]. Employees with proactive personalities tend to engage in initiatives and change the environment to achieve their goals [26,27,28]. Proactive employees were found to have better person-organization (P-O) and person-job (P-J) fit than passive employees. P-O fit is the convergence between individual and organizational values [29], and P-J fit is the convergence between employee capabilities and job demands [30]. For example, proactive employees enhance their task mastery for a better future [31], and to be consistent with their preferences, proactive employees adjust their work settings or procedures [26,32].

Perceived work competence is the self-perceived ability of an employee to perform tasks with skill in the workplace [33]. More specifically, an employee’s perceived work competence can be defined by the knowledge, skills, and judgment the employee uses to complete work activities [34]. Because proactive people tend to seek opportunities, take action, persevere until they bring about changes and meet the desired goals [35], and judge highly their capabilities to handle the various situations that arise during practical work, therefore, we hypothesize the following:

**Hypothesis** **1.** *Proactive personality has a positive influence on perceived work competence.*

### 1.3. The Mediating Role of Work Engagement

Schaufeli et al. [24]. defined work engagement as “a positive, fulfilling, work-related state of mind that is characterized by vigour, dedication, and absorption”. Numerous studies have not only consistently shown that proactive personality and work engagement are positively correlated [36,37,38,39] but also highlighted that proactive personality might be an antecedent of work engagement [40]. According to the JD-R model, job resources encompass “physical, psychological, social or organizational aspects of the job”, which support employees to achieve their job tasks [15]. To bring about meaningful changes, employees with proactive personalities are known to be good at identifying and responding to opportunities by utilizing available resources in the workplace [36,41,42].

Furthermore, employees who are more engaged in their work are also likely to experience greater personal accomplishment. Researchers have argued that perceived work competence is closely related to personal accomplishment, which is the feeling of productivity and effectiveness at work [43,44]. In other words, more engaged employees enjoy the feeling that they are competent through executing work tasks productively and effectively. Taken together, when proactive individuals are relatively unconstrained by situational hindrances, they are more willing to involve themselves in work, which in turn enhances their sense of perceived work competence. Subsequently, we propose the following hypothesis:

**Hypothesis** **2.** *Work engagement positively mediates the relationship between proactive personality and perceived work competence.*

### 1.4. The Moderating Role of Perceived Supervisor Support

Perceived supervisor support is the extent to which workers perceive that their supervisor cares about their well-being and values their contributions [45]. Drawing on the JD-R model, job resources, such as a supportive environment for workers, have been considered to both extrinsically and intrinsically energize employees to facilitate the accomplishment of the required tasks. For example, perceived supervisor support empowers employees to develop and improve their general job performance [46] and increases their motivation [18] and interest [47] in the assigned tasks. There is wide research indicating that the extent to which employees perceive support from the workplace influences their engagement at work [48,49,50]. Hence, we propose the following hypothesis:

**Hypothesis** **3.** *Perceived supervisor support moderates the relationship between proactive personality and work engagement. When perceived supervisor support is high, the relationship between proactive personality and work engagement is strengthened.*

Furthermore, employees perceive themselves as more self-competent when they immerse themselves in the personal accomplishment derived from utilizing their knowledge, skills, and abilities at work [51]. Therefore, based on the JD-R model, supervisor support, as a job resource, could stimulate employees’ personal growth and development and reinforce their perceptions of work competence when they are more engaged at work [15]. This would strengthen the links between employee engagement and positive self-perceptions and self-value. Therefore, we propose the following:

**Hypothesis** **4.** *Perceived supervisor support moderates the relationship between work engagement and perceived work competence. When perceived supervisor support is high, the relationship between work engagement and perceived work competence is strengthened.*

### 1.5. The Moderated Mediating Effect of Supervisor Support

From a psychological resource perspective, work engagement emphasizes energy and resilience at work, as well as demonstrating effort and perseverance [49]. These psychological resources are also salient for proactive employees who are unrestricted by situational hindrances, persist until the change is achieved, and are more inclined to engage at work to achieve goals. Additionally, supervisor support provides intrinsic and extrinsic motivation, which fortifies the motivational process of the JD-R model. The more proactive employees feel they receive assistance and reassurance from their supervisors, the more likely they are to enhance their work engagement and foster perceived work competence. Based on the discussion proposed above, we formulate the following moderated mediation hypothesis:

**Hypothesis** **5.** *In the mediating model of work engagement, perceived supervisor support simultaneously moderates the indirect effects of proactive personality on perceived work competence, which would be strengthened.*

## 2. Materials and Methods

### 2.1. Participants and Procedures

The participants were sports coaches at all education levels in public schools in Taiwan. Convenience sampling was used to recruit participants for this study. All participants volunteered and provided informed consent. Of the 261 participants, 70.5% were male, the mean age was 38.5 years (SD = 9.23), and the mean tenure was 10.13 years (SD = 8.31). Most of the participants had a bachelor’s degree (59.4%).

### 2.2. Measures

Since there are no Chinese versions of the work engagement and perceived work competence scales, we used a back-translation step [52] to develop Chinese versions of these scales. First, two bilingual experts translated the instrument from English to Chinese. Second, back-translation steps were taken to ensure that the translation did not deviate from the meaning of the original measure. Each scale is scored using a Likert-type scale, with 1 to 7 representing “strongly disagree” to “strongly agree.”

#### 2.2.1. Proactive Personality

We assessed proactive personality by using the Chinese version of Lu and Kuo’s [53] Proactive Personality Scale with 4 items. The Cronbach’s alpha was 0.83.

#### 2.2.2. Work Engagement

We assessed work engagement by using Schaufeli et al.’s [54] Work Engagement Scale, which includes 12 items. The Cronbach’s alpha was 0.97.

#### 2.2.3. Perceived Work Competence

We assessed perceived work competence by using Warr’s [55] Perceived Work Competence Scale, which included 6 items. The Cronbach’s alpha was 0.83.

#### 2.2.4. Perceived Supervisor Support

We assessed perceived supervisor support by using the Chinese version of Chien et al.’s [56] supervisor support scale, which has 4 items. The Cronbach’s alpha was 0.96.

#### 2.2.5. Control Variables

Researchers have indicated that sex, age, and education level may be connected with proactive personality and work engagement [57]. Additionally, studies have shown that background variables such as tenure, age, and education level are related to supervisor support [58]. Therefore, sex, age, education level, and tenure were the control variables in this study.

## 3. Results

### 3.1. Descriptive Analyses

In Table 1, for focal variables, proactive personality, work engagement, perceived work competence, and perceived supervisor support were all positively correlated with each other (*ps* < 0.05).

### 3.2. Proactive Personality and Perceived Work Competence

We tested the effects of proactive personality on perceived work competence (i.e., Hypothesis 1) with the 1st step in Baron and Kenny’s [59] procedure for mediation examination with four steps. As shown in Table 2 (Model 4), the overall model was significant, *F* (5, 255) = 15.60 (*p* < 0.05). Proactive personality (*β* = 0.40, *p* < 0.05) positively influenced perceived work competence. Hence, Hypothesis 1 was supported.

### 3.3. The Mediating Role of Work Engagement

In Table 2 (Model 2), with the 2nd step of Baron and Kenny’s [54] procedure, the overall model was significant, *F* (5, 255) = 16.92, *p* < 0.05), and proactive personality significantly predicted work engagement (*β* = 0.38, *p* < 0.05). Then, following the 3rd step of Baron and Kenny’s [59] procedure (Model 6), the overall model was significant (*F* (6, 254) = 38.82, *p* < 0.05), and work engagement (*β* = 0.57, *p* < 0.05) significantly predicted perceived work competence after controlling for proactive personality. Finally, in the 4th step, compared to the effect of proactive personality on perceived work competence in the 1st step (*β* = 0.40, *p* < 0.05), the effect of proactive personality on perceived work competence in the 3rd step (*β* = 0.18, *p* < 0.05) was significantly decreased. Thus, Hypothesis 2 is supported.

### 3.4. The Moderating Role of Supervisor Support

Table 3 displays the moderating effects of perceived supervisor support on the relationship between proactive personality and work engagement. In Model 2, the results showed that both proactive personality (*β* = 0.33, *p* < 0.05) and perceived supervisor support (*β* = 0.30, *p* < 0.05) significantly predicted work engagement. In Model 3, after controlling for the control variables, proactive personality, and perceived supervisor support, the interaction term (*β* = −0.15, *p* < 0.05) provided significantly incremental explained variance on work engagement.

**Figure 2:**
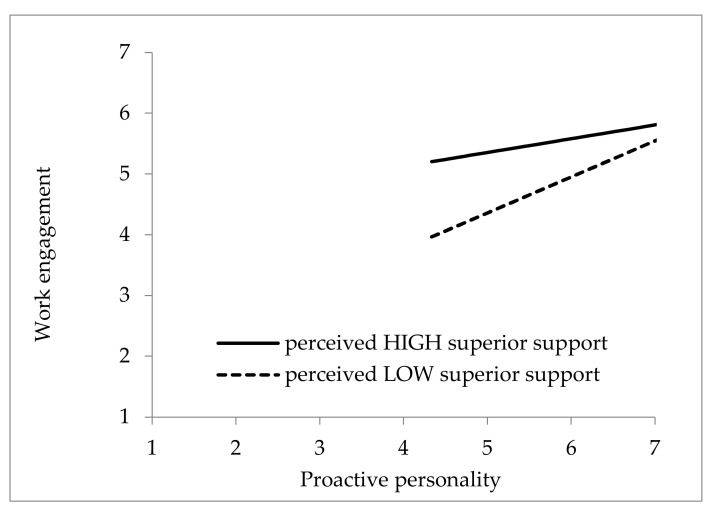
The interaction effect of proactive personality and perceived supervisor support on work engagement.

The interaction effect is shown in Figure 2 with 1 SD above and below the means of perceived supervisor support. Figure 2 shows that proactive personality had a stronger positive association with work engagement when perceived supervisor support was low than when it was high. These findings partially support Hypothesis 3. Supervisor support moderates the link between proactive personality and work engagement. However, the positive relationship between proactive personality and work engagement is strengthened when sports coaches perceive a low level of supervisor support.

Table 4 displays the moderating effects of perceived supervisor support on the relationship between work engagement and perceived work competence. In Model 2, the results showed that only work engagement (*β* = 0.65, *p* < 0.05) significantly predicted perceived work competence. In Model 3, after controlling for the main effects, the interaction term (*β* = 0.11, *p* < 0.05) significantly explained the incremental variance of perceived work competence.

**Figure 3:**
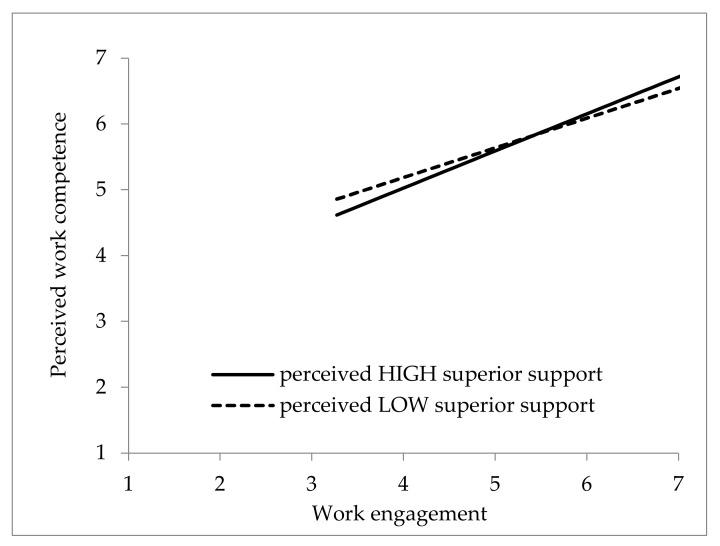
Interaction effect of work engagement and perceived supervisor support on perceived work competence.

The interaction effect is shown in Figure 3 with 1 SD above and below the means of perceived supervisor support. Figure 3 shows that work engagement had a stronger positive association with perceived work competence when perceived supervisor support was high than when it was low. These findings supported Hypothesis 4 that the positive relationship between work engagement and perceived work competence is strengthened when sports coaches perceive a high level of supervisor support.

### 3.5. Moderated Mediation Analyses

We used Haye’s [60] PROCESS syntax of Model 58 to examine our proposed moderated mediation model. We examined the conditional indirect effect of proactive personality on perceived work competence through work engagement at three levels of perceived supervisor support: M, M + 1 SD, and M − 1 SD [61]. Through 1000 times bootstrapping, the results indicated that when sports coaches perceived low supervisor support, the indirect effect of proactive personality on perceived work competence through work engagement was stronger (effect size = 0.26, 95%, confidence interval = [0.11, 0.39]) than when they perceived high supervisor support (effect size = 0.14, 95%, confidence interval = [0.02, 0.29]). In other words, when perceived supervisor support was low, the above indirect effects were stronger. These findings partially support Hypothesis 5. Supervisor support simultaneously moderated the indirect effects of proactive personality on perceived work competence via work engagement; however, the indirect effects were strengthened only when sports coaches perceived low supervisor support.

In conclusion, the results of our hypothesis were summarized as the following:

Hypothesis 1 is supported. When sports coaches would have a higher proactive personality, they have better-perceived work competence.

Hypothesis 2 is supported. When sports coaches would have a higher proactive personality, they have better-perceived work competence through higher work engagement.

Hypothesis 3 is partially supported. Perceived supervisor support moderates the relation between sports coaches’ proactive personality and their work engagement. However, the positive relationship between proactive personality and work engagement would be strengthened when sports coaches perceive a low level rather than a high level of supervisor support.

Hypothesis 4 is supported. Perceived supervisor support moderates the relationship between sports coaches’ work engagement and their perceived work competence, and this link would be strengthened when perceived supervisor support is high.

Hypothesis 5 is partially supported. In the mediating model of work engagement, perceived supervisor support simultaneously strengthens the indirect effects of the sports coaches’ proactive personality on their perceived work competence; however, this is when perceived supervisor support is at low instead of high levels.

## 4. Discussion

To advance our knowledge of the predicting variables of school sports coaches’ perceived work competence, we constructed a conceptual model based on the JD-R model to validate the mediating effect of work engagement in the relationship between proactive personality and perceived work competence and determined how these variables impacted their perceived work competence. In addition, our study further tested perceived supervisor support as a moderator to investigate the relationships among proactive personality, work engagement, and perceived work competence. It was found that, as the theory and hypothesis assumed, work engagement partially mediated the impact of proactive personality on perceived work competence. The proactive personality promotes work engagement, which in turn positively increases employees’ perceived work competence. The results also revealed that perceived supervisor support moderates the relationships between proactive personality and work engagement. However, the current results partially support the moderating effects of Hypothesis 3 and 5. It showed that when sports coaches perceive a low level of supervisor support, the positive relationship between proactive personality and work engagement is strengthened. In addition, when perceiving low supervisor support, their proactive personality triggered work engagement, which in turn increased their perceived work competence.

Based on the JD-R model literature, our findings show that dispositional proactive personality is positively related to work engagement, which is in turn positively related to perceived work competence. This finding addresses Xanthopoulou et al.’s [62] concern about the role of personal resources as the antecedents of work engagement and their respective outcomes in the JD-R model. Consistent with past studies [36,63], our findings also support that a proactive personality is a relevant predictor of work engagement. Proactive individuals demonstrate initiative and perseverance [64], which is likely related to immersing themselves in their work [35]. Since work engagement covers the basic dimensions of intrinsic motivation, such as vigor, dedication, and absorption [65], there could be expected to be a mediational construct that links personal assertiveness and perceived work competence. Sports coaches with highly proactive personalities are more likely to engage in their jobs and further experience a high level of perceived work competence from the value of work outcomes. In conclusion, the present study proposes that personal resources such as a proactive personality play a significant antecedent role in the JD-R model; proactive sports coaches may engage more in their work, thereby increasing their perceptions of perceived work competence.

Our findings show that perceived superior support moderates the relationship between proactive personality and work engagement. However, the result did not correspond exactly to that of Hypothesis 3, which showed that when the sports coaches perceived low superior support, the relationship between proactive personality and work engagement was strengthened. According to past studies, we found inconsistent results when discussing the moderating role of perceived social support. Illustration has been found that high social support may weaken the positive effect of a proactive personality on safety behaviors [66]. On the contrary, another study has revealed that under high levels of perceived social support, a proactive personality can more effectively reduce college students’ career decision-making difficulties [67]. It is argued that the moderating effect of social support needs further validation, especially when interacting with a proactive personality. According to a past study [68], employees who perceive high supervisor support may benefit from fair treatment, reward recognition, and proper guidance. In contrast, employees who perceive low superior support may view their workplaces as having a risky climate in which they feel they are untrusted and worthless. Under such constrained circumstances, proactive employees may persevere in the face of predicaments and tend to be more effective in seeking better solutions and engaging more to improve the outcomes of their tasks [11,32]. Proactive people tend to expand their role requirements and surpass the expectations of their formal responsibilities for the purpose of influencing their work environment [69]. As a result, when sports coaches perceive less support from their superiors, their proactive personalities may drive them to seek better solutions and more fully engage in their tasks to overcome difficulties.

In addition, our findings also show that perceived superior support moderates the relationship between work engagement and perceived work competence. The results reveal that work engagement is positively related to perceived work competence, particularly when sports coaches perceived high superior support. There is substantial research indicating that employees who perceive support in their workplaces have better work engagement [48]. Studies have highlighted that perceived supervisor support increases employees’ motivation for assigned tasks and that these employees empower themselves to develop and improve their job performance [70]. In sports settings, perceived supervisor support also plays an important role in predicting sports coaches’ work outcomes [71]. Because the constructive feedback from superiors and the decision-making control that is given to employees allows them to experience autonomy and competence [72], support from supervisors should be a target for enhancement to foster the relationship between coaches and their respective organizations [71]. In summary, sports coaches who perceive high supervisor support may benefit from constructive feedback and autonomy, which facilitate better connection with their work engagement and perceived work competence.

Our study further demonstrates a moderated mediation with perceived supervisor support as the moderator to investigate the relationships among proactive personality, work engagement, and perceived work competence. The results reveal that under low supervisor support, sports coaches with high proactive personalities exhibit higher work engagement, which in turn improves their perceived work competence. A past study also reported that employees characterized by a proactive personality were most likely to craft their jobs, which in turn was predictive of work engagement and in-role performance [36]. Conceptually, the findings of the current study are consistent with Daniels’s [73] claim that the enactment of employees’ tendencies is the most important predictor of performance in an organizational context. Since proactive individuals often seize opportunities, show initiative, take action, and persevere until they reach closure by bringing about changes [26], they are relatively unconstrained by situational conditions. As a result, even when proactive sports coaches perceive low support from their directors, they can show more initiative and take action until they bring about changes to the constrained situation. The more they engage in their tasks, the greater the possibility they overcome these difficulties and achieved higher perceived work competence.

### 4.1. Theoretical and Practical Implications

The main theoretical implication of the current study is to clarify the role of proactive personality as a personal resource and as a vital antecedent in the motivation process of the JD-R model. Although the positive effects of proactive personality on work engagement have been revealed in recent research [40], little work has been done to examine the relationship between proactive personality and perceived work competence via work engagement. The current findings suggest that a proactive personality can trigger sports coaches’ work engagement and further improve their perceived work competence, even when perceiving low support from superiors. The unique function of proactive personality in the sport setting is validated. Future studies should contribute by exploring other potential personal resources as antecedents of sports coaches’ work behavior. Moreover, the other important theoretical implication of the current study is that perceived superior support plays an important role in interpreting the boundary conditions of the JD-R model and further contributes to the interaction effect in predicting perceived work competence. Future research should incorporate other personal and situational factors to examine the dynamic process of predicting in-role and extra-role work performance.

The current study also provided some practical insight. Because proactive personality exerts a great function in predicting work engagement as well as the perceived work competence of sports coaches, it is relatively important to enhance their proactive thinking through training. Following a proactivity training intervention [74], sports coaches may facilitate their ability to recognize opportunities, defend against threats, and leverage core competencies into competitive advantage [74]. It could be an effective technique for schools, organizations, and the government to improve sports coaches’ productivity and competitive advantage. In addition, our study also illustrated the importance of superior support in school settings. According to O’Driscoll and Beehr [75], supervisors are the most salient individuals in employees’ working contexts. Employees who perceive support from their organizations tend to be very inspired, more spirited, and have a positive attitude in the workplace [76]. School administrators should provide more tangible support for sports coaches, such as well-defined guidance and requisite backups. Under adequate support from superiors, sports coaches could exert more effort in their jobs and gain higher work competence.

### 4.2. Limitations and Future Suggestions

There are several limitations when interpreting the results. First, our study used a cross-sectional design to collect data, which may have inflated the correlations among variables. Future studies might adopt a longitudinal design to measure the predictor variables in the first wave, collect the mediator and moderator in the second wave, and collect work and psychological outcome data in the third wave. It may also help better study interval changes in a construct [77] and avoid inflations between variables. Second, our study adopted only self-report measures to assess the antecedents, mediators, moderators, and outcomes. Although we used reliable and valid measures that were not conceptually confounded, there was still a potential threat of common method variance [78]. Future research using multiple-resource methods, such as perceived work competence evaluated by peers or superiors, is recommended. Finally, in the current study, we recruited sports coaches from public schools as participants and did not include coaches of professional teams or club sports. Indeed, there is a substantial difference between schools and profit organizations when discussing the workplace environments of sports coaches. To further contribute to JD-R theory and application, there is a need to investigate the working context of sports coaches of professional teams and clubs, which may help enrich the growing body of sports coach research.

## Figures and Tables

**Table 1:** Means, standard deviations, and correlations among variables (*n* = 261).

Variable	M	SD	1	2	3	4	5	6	7
1. Sex	-	-							
2. Age	39.85	9.06	−0.17 *						
3. Education level	2.36	0.53	0.08	0.10					
4. Tenure	10.13	8.31	−0.02	0.76 *	0.09				
5. Proactive personality	5.90	0.78	−0.02	0.02	−0.06	0.04			
6. Work engagement	5.51	1.12	−0.21 *	0.25 *	−0.04	0.24 *	0.40 *		
7. Perceived work competence	5.80	0.82	−0.20 *	0.19 *	−0.00	0.19 *	0.41 *	0.67 *	
8. Perceived supervisor support	5.40	1.51	−0.03	−0.11	−0.13 *	−0.10	0.20 *	0.34 *	0.22 *

Note: M = Mean, SD = Standard deviation. * *p* < 0.05.

**Table 2:** Mediation analysis.

Variable	Work Engagement		Perceived Work Competence			
	Model 1	Model 2	Model 3	Model 4	Model 5	Model 6
Sex	−0.19 *	−0.18 *	−0.19 *	−0.19 *	−0.07	−0.08
Age	0.08	0.09	0.03	0.04	−0.02	−0.01
Education level	−0.05	−0.03	0.00	0.02	0.03	0.04
Tenure	0.18	0.15	0.17	0.14	0.05	0.05
Proactive personality		0.38 *		0.40 *		0.18 *
Work engagement					0.65 *	0.57 *
*R* ^2^	0.10	0.25	0.08	0.23	0.45	0.48
*Adj R* ^2^	0.09	0.23	0.06	0.22	0.44	0.47
*F*	7.41 *	16.92 *	5.30 *	15.60 *	41.95 *	38.82 *
*df*	4, 256	5, 255	4, 256	5, 255	5, 255	6, 254

* *p* < 0.05.

**Table 3:** Hierarchical regression analysis of study variables and their interaction with work engagement.

Variable	Work Engagement		
	Model 1	Model 2	Model 3
Sex	−0.19 *	−0.17 *	−0.17 *
Age	0.08	0.12	0.12
Education level	−0.05	0.00	0.01
Tenure	0.18	0.17 *	0.17 *
Proactive personality		0.33 *	0.29 *
Perceived supervisor support		0.30 *	0.30 *
Proactive personality × Perceived supervisor support			−0.15 *
*R* ^2^	0.10 *	0.33 *	0.35 *
Δ*R*^2^		0.23 *	0.02 *

* *p* < 0.05.

**Table 4:** Hierarchical regression analysis of variables and their interaction with perceived work competence.

Variable	Perceived Work Competence		
	Model 1	Model 2	Model 3
Sex	−0.19 *	−0.07	−0.07
Age	0.03	−0.02	−0.02
Education level	0.00	0.03	0.03
Tenure	0.17	0.05	0.05
Work engagement		0.65 *	0.69 *
Perceived supervisor support		0.00	0.01
Work engagement × Perceived supervisor support			0.11*
*R* ^2^	0.08 *	0.45 *	0.46 *
Δ*R*^2^		0.38 *	0.01 *

* *p* < 0.05.

## Data Availability

The datasets used and analyzed in the current study are available from the corresponding author upon reasonable request.
